# Prenatal and birth predictors of objectively measured physical activity and sedentary time in three population-based birth cohorts in Brazil

**DOI:** 10.1038/s41598-019-57070-x

**Published:** 2020-01-21

**Authors:** Ding Ding, Gregore I. Mielke, Inacio Crochemore M. Silva, Fernando C. Wehrmeister, Bernardo L. Horta, Soren Brage, Pedro C. Hallal, Ulf Ekelund

**Affiliations:** 1 Prevention Research Collaboration, School of Public Health, and Charles Perkins Centre, University of Sydney, Camperdown, NSW 2006 Australia; 2 Postgraduate Program in Epidemiology, Federal University of Pelotas, Brazil Rua Marechal Deodoro, 1160 - 3° Piso, Bairro Centro, Pelotas, RS Cep: 96020-220 Caixa Postal 464, Brazil; 3 School of Human Movement and Nutrition Sciences, University of Queensland, St Lucia, QLD 4067 Australia; 4 Postgraduate Program in Epidemiology and International Center for Equity in Health, Federal University of Pelotas, Rua Marechal Deodoro, 1160 - 3° Piso, Bairro Centro, Pelotas, RS Cep: 96020-220 Caixa Postal 464, Brazil; 5 MRC Epidemiology Unit, University of Cambridge School of Clinical Medicine, Cambridge, CB20SL UK; 6 Norwegian School of Sports Sciences, PO Box 4014, Ulleval Stadion 0806, Oslo, Norway

**Keywords:** Epidemiology, Risk factors

## Abstract

Physical inactivity is a global pandemic with no signs of improvement. Prolonged sitting time is an emerging risk factor that exacerbates the health consequences of physical inactivity. Both behaviours are influenced by various individual and environmental factors but it remains unknown whether early-life exposures “program” these behaviours in later life. The current evidence is limited by a small number of studies which were primarily conducted in high-income countries, and a narrow range of early-life variables examined. Using data from three population-based Brazilian birth cohorts (analytical samples: n = 2740 for 1982 cohort, aged 30 years; n = 3592 for 1993 cohort, aged 18; n = 2603 for 2004 cohort, aged 6), we show that being female and higher family socioeconomic status at birth are strong and consistent predictors of lower physical activity and higher sedentary time from childhood to adulthood. Meanwhile, higher birth weight and lower birth order may also predict lower physical activity and higher sedentary time. Our findings are distinct from evidence from high-income countries, suggesting the importance of broader socioeconomic context in determining individual’s activity patterns through the life- course. Such evidence is essential for understanding the biological etiology and socioeconomic context of physical activity and sedentary behaviour at an early stage in life.

## Introduction

Physical inactivity is responsible for chronic diseases, premature mortality^1^ and substantial economic burdens^2^. With one in three adults and four in five adolescents worldwide failing to achieve the recommended levels of physical activity^3^ and no signs of improvement at the population level^4^, the pandemic of physical inactivity is one of the biggest challenges to global health. Sedentary behaviour, on the other hand, is a distinct class of waking behavior in a sitting, reclining, or lying posture with an energy expenditure ≤1.5 Metabolic Equivalents^5^. Increasing evidence suggests that prolonged sedentary time may elevate the risk of chronic diseases and mortality in adults and the combination of prolonged sitting time and low levels of physical activity may be particularly detrimental to health^6^.

Understanding the causes of physical inactivity and prolonged sedentary time is essential for developing interventions to improve public health. Hence, many studies have been conducted to understand why some people are more active than others. It is generally understood that physical activity and sedentary behaviour are jointly influenced by a range of individual, interpersonal, environmental and policy factors across different stages in life^7,8^. However, one consistently understudied area is whether and how prenatal, birth and early life factors influence these behaviours^9,10^. The Developmental Origins of Health and Disease Theory, based on Barker’s hypothesis^11^, emphasises the important roles of non-optimal growth and early life environments in “programming” health and disease outcomes in utero and during early life^12^. While most studies focused on early “programming” of physiological outcomes, such as adiposity and hypertension^13^, few explored behavioral outcomes^14^. Given that physical activity and sedentary behaviour are habitual in nature and can track from childhood to adulthood^15,16^, it is logical to hypothesize that some of their determinants may manifest early in life, potentially before or at birth^9^. Indeed, animal studies suggest a biologically plausible programming effect of physical activity and sedentary behaviour^17,18^. These studies suggest that an unfavourable, under- or over-nourished prenatal environment appears to program lower levels of spontaneous physical activity, persistent into adulthood^18^.

To date, a small number of epidemiological studies have examined the prenatal and birth predictors of physical activity and sedentary behaviour with the primary focus on birth weight and gestational age. Findings from these studies suggest that birth weight is likely to have a weak association with physical activity in childhood, adolescence^19^, and adulthood^20^, but there is little evidence for an association of birth weight and gestational age with sedentary behaviour among young people^9^. A broader range of predictors, such as maternal weight status, birth order and type of delivery, have been less explored.

Furthermore, given that most studies on early life predictors of physical activity and sedentary behaviour to date were conducted in high-income countries^9,19^, and that childhood socioeconomic status plays an important role in later physical activity^21^, it is important to understand early life predictors of both biological and socioeconomic nature in a low- and middle- income country setting. Building such evidence is essential for understanding the biological etiology and socioeconomic context of physical activity and sedentary behaviour and for identifying and targeting those who may be at risk of an inactive lifestyle at an early stage in life.

To address these research gaps, we used data from three population-based birth cohorts of children and adults from Pelotas, Brazil, where rapid economic, lifestyle and epidemiological transitions were observed in the past few decades. We aimed to examine the association between a series of prenatal and birth predictors and objectively measured physical activity and sedentary behaviour later in life during childhood, adolescence and adulthood.

## Results

This study is based on the 1982, 1993, and 2004 Pelotas Birth Cohorts. Pelotas is located in the Southern state of Rio Grande do Sul. These cohorts were comprised of live births, whose families lived in the urban area of Pelotas, delivered at maternity hospitals during the year of data collection. In 1982, 1993 and 2004, 6011, 5265, 4263 live births were reported in Pelotas, and 5914, 5249, 4231 were recruited to the cohort, representing >98% of all live births in respective years in the city. Infants were measured, and their mothers interviewed within 24 hours of delivery. Prenatal and birth variables include those of both biological (i.e., sex, birth weight, gestational age, birth order, mode of delivery, and pre-pregnancy body mass index [BMI]) and socioeconomic nature (i.e., maternal education and family income). Cohort participants have been followed up at different times since birth. The present analyses are based on baseline and follow-up accelerometry data collected in 2010–2013 when participants were aged 30, 18, and 6 years, respectively. Three outcome variables were derived from accelerometry data: (1) measured physical activity of any intensity, which approximates the overall physical activity volume (milli-g), (2) moderate-to-vigorous intensity physical activity ([MVPA]min/day), which is conventionally considered activities within the “health enhancing” intensity threshold and a key component of physical activity guidelines, (3) sedentary time (min/day), which quantifies the total amount of sedentary behaviour.

The final analytical samples included 2740 participants from the 1982 cohort, 3592 from the 1993 cohort, and 2603 from the 2004 cohort, representing 46%, 68%, and 62% of the baseline participants (Supplementary Fig. 1). As shown in Table 1, females accounted for slightly more than half of the 1982 and 1993 cohorts and less than half of the 2004 cohort. The distribution of birth weight was similar across cohorts with 7–9% classified as low birthweight. Compared with the 1982 cohort, there was a higher proportion of preterm births and cesarean deliveries in the later cohorts. A lower proportion of mothers were classified as underweight and a higher proportion overweight or obese in the 2004 cohort compared with earlier cohorts. Across all cohorts, around 40% of the participants were first-borns, and more than 40% were second- or third-born. The distribution of family income were similar across cohorts with the majority reporting a family income of ≤3 times the minimum wage. The proportion of mothers with nine years of schooling or more was considerably higher in the 2004 cohort. The 2004 cohort had the highest levels of overall and moderate-to-vigorous physical activity and the lowest level of sedentary time (Table 1). Characteristics of the overall baseline samples and the analytical samples are comparable (Supplementary Table 1).

**Table 1:** Characteristics of the three birth cohorts in Pelotas, Brazil.

	1982 Cohort	1993 Cohort	2004 Cohort
	(n = 2740)*	(n = 3592)*	(n = 2603)*
Age (years: mean[SD])	30.2 (0.2)	18.4 (0.3)	6.7 (0.3)
Characters at birth: n (%)			
Family income (No. of minimum wages per month)			
≤1	588 (21.6)	636 (17.1)	524 (20.1)
1.1–3.0	1383 (50.7)	1557 (43.4)	1204 (46.3)
3.1–6.0	505 (18.5)	866 (24.1)	599 (23.0)
6.1–10.0	141 (5.2)	286 (8.0)	154 (5.9)
>10	109 (4.0)	247 (6.9)	121 (4.7)
Maternal education (years of schooling)			
0	140 (5.1)	85 (2.4)	19 (0.7)
1–4	791 (28.9)	879 (24.5)	365 (14.2)
5–8	1215 (44.4)	1716 (47.8)	1092 (42.4)
9+	590 (21.6)	909 (25.3)	1100 (42.7)
Sex			
Male	1329 (48.5)	1764 (49.1)	1341 (51.5)
Female	1411 (51.5)	1828 (50.9)	1262 (48.5)
Pre-pregnancy body mass index (kg/m^2^)			
<18.5	176 (7.5)	309 (8.8)	78 (4.3)
18.5–24.9	1625 (69.5)	2373 (67.8)	1071 (59.2)
25–29.9	421 (18.0)	645 (18.4)	446 (24.7)
≥30	116 (5.0)	172 (4.9)	213 (11.8)
Birth order			
1	1080 (39.4)	1426 (39.7)	1159 (45.3)
2 or 3	1218 (44.5)	1635 (45.6)	1060 (41.4)
≥4	441 (16.1)	528 (14.7)	339 (13.3)
Gestational age (weeks)			
<37	120 (5.5)	381 (10.7)	338 (13.0)
≥37	2075 (94.5)	3171 (89.3)	2263 (87.0)
Type of delivery			
Vaginal	2013 (73.5)	2474 (68.9)	1429 (54.9)
Cesarean	727 (26.5)	1118 (31.1)	1174 (45.1)
Birth weight (g)			
<2500	196 (7.2)	331 (9.2)	224 (8.6)
2500–3499	1711 (62.4)	2285 (63.7)	1663 (63.9)
≥3500	832 (30.4)	972 (27.1)	716 (27.5)
Accelerometer outcomes			
Overall physical activity (milli-g)	35.5 (10.5)	39.3 (12.9)	59.4 (16.3)
Total moderate-to-vigorous physical activity (minutes/day)	25.8 (31.4)	43.0 (42.2)	44.6 (34.7)
Total sedentary time (minutes/day)	679.6 (88.8)	688.5 (90.5)	607.1 (74.0)

*Numbers vary owing to missing values.

Across all three cohorts and outcomes, unadjusted and adjusted analyses yielded similar patterns of associations except for an attenuation of effect sizes in adjusted models in most cases. Results described below are primarily based on those from adjusted analyses.

With respect of overall physical activity, males and those with lower family income and maternal education at birth had higher overall physical activity (Table 2). Specifically, the average difference between male and female participants ranged between 5.1 and 9.2 milli-g across cohorts, with the difference between sex being the biggest in the youngest cohort, and the smallest in the oldest cohort. The association between maternal education levels and overall physical activity showed a dose-repose pattern with the average difference between the lowest and highest education categories being around 6.5 milli-g for the 2004 cohort, 5.3 milli-g for the 1993 cohort, and 3.1 milli-g for the 1982 cohort. The association between family income and overall physical activity was less dose-response, however, the lowest income category had consistently the highest levels of physical activity, which was around 2 milli-g higher than the highest income group across all three cohorts. Being born preterm was associated with slightly more overall physical activity (i.e., 2.0 mili-g) in the 1982 cohort only. Cesarean delivery was significantly associated lower overall physical activity in unadjusted models but was only statistically significantly associated with physical activity in the adjusted model in the 1982 cohort; however, the average difference was only around 1.0 mili-g. Lower birth weight and higher birth order were associated with higher levels of physical activity in the 1993 and 2004 cohorts in the adjusted models. Specifically, the association between birthweight and overall physical activity was the strongest in the 1993 cohort where the average difference between the lowest and highest birthweight category was 3.8 mili-g. In terms of birth order, the difference between firstborns and those with the highest birth order (i.e., 4+) was around 3 mili-g in both cohorts.

**Table 2:** Unadjusted and adjusted associations^a^ between predictors at birth and overall physical activity (mean acceleration^b^) in three birth cohorts in Pelotas, Brazil.

Variables	1982 Cohort (n = 2740)^c^				1993 Cohort (n = 3592)^c^				2004 Cohort (n = 2603)^c^			
	Unadjusted β (95% CI)	p	Adjusted β (95% CI)	p	Unadjusted β (95% CI)	p	Adjusted β (95% CI)	p	Unadjusted β (95% CI)	p	Adjusted β (95% CI)	p
Family income (No of minimum wages per month)		<0.001		0.027		<0.001		<0.001		<0.001		0.009
≤1	5.33 (3.20; 3.47)		2.01 (−0.29; 4.30)		6.02 (4.15; 7.89)		2.25 (0.32; 4.18)		4.89 (1.69; 8.09)		2.36 (−0.94; 5.67)	
1.1–3.0	4.54 (2.48; 6.57)		1.85 (−0.29; 3.99)		5.18 (3.47; 6.89)		2.25 (0.51; 3.98)		2.94 (−0.09; 5.97)		0.89 (−2.21; 3.99)	
3.1–6.0	2.24 (0.07; 4.41)		0.59 (−1.56; 2.74)		1.98 (0.19; 3.79)		0.09 (−1.67; 1.85)		0.48 (−2.68; 3.64)		−0.63 (−3.77; 2.50)	
6.1–10.0	1.95 (−0.67; 4.56)		1.03 (−1.49; 3.56)		0.40 (−1.18; 2.57)		−0.29 (−2.32; 1.75)		−0.28 (−4.14; 3.57)		−0.77 (−4.52; 2.99)	
>10	Ref		Ref		Ref		Ref		Ref		Ref	
Maternal education (year of schooling)		<0.001		<0.001		<0.001		<0.001		<0.001		0.001
0	4.00 (2.08; 5.91)		3.06 (0.99; 5.14)		7.65 (4.77; 10.54)		5.31 (2.51; 8.11)		8.12 (0.78; 15.46)		6.53 (−0.58; 13.65)	
1–4	4.60 (3.49; 5.71)		3.32 (2.01; 4.63)		6.00 (4.82; 7.17)		3.89 (2.63; 5.16)		4.73 (2.81; 6.64)		2.62 (0.52; 4.73)	
5–8	3.74 (2.72; 4.76)		2.75 (1.60; 3.89)		4.43 (3.41; 5.45)		2.97 (1.92; 4.03)		3.50 (2.15; 4.86)		2.30 (0.86; 3.75)	
9+	Ref		Ref		Ref		Ref		Ref		Ref	
Sex		<0.001		<0.001		<0.001		<0.001		<0.001		<0.001
Male	Ref		Ref		Ref		Ref		Ref		Ref	
Female	−5.08(−5.85; −4.31)		−5.14 (−5.89; −4.38)		−8.50 (−9.30; −7;70)		−8.59 (−9.37; −7;80)		−9.06 (10.26; −7.86)		−9.19 (−10.40; −7.98)	
Pre-pregnancy body mass index (kg/m^2^)		0.056		0.039		0.063		0.245		0.462		0.523
<18.5	1.39 (−0.26; 3.05)		0.90 (−0.66; 2.46)		2.25 (0.73; 3.77)		1.01 (−0.58; 2.27)		−3.77 (−7.52; −0.01)		−1.99 (−5.29; 1.31)	
18.5–24.9	Ref		Ref		Ref		Ref		Ref		Ref	
25–29.9	−0.80 (−1.94; 0.34)		−0.90 (−2.00; 0.20)		0.07 (−1.04; 1.19)		0.10 (−0.94; 1.14)		−0.19 (−2.00; 1.61)		−0.14 (−1.77; 1.50)	
≥30	−0.24 (−2.24; 1.76)		−0.78 (−2.69; 1.13)		−0.44 (−2.43; 1.55)		−0.73 (−2.58; 1.13)		0.03 (−2.37; 2.43)		0.27 (−2.05; 2.60)	
Birth order		0.020		0.113		<0.001		<0.001		<0.001		0.015
1	Ref		Ref		Ref		Ref		Ref		Ref	
2 or 3	0.63 (−0.23; 1.49)		0.65 (−0.19; 1.49)		1.41 (0.50; 2.32)		1.10 (0.25; 1.96)		0.86 (−0.49; 2.21)		0.81 (−0.54; 2.15)	
≥4	1.33 (0.17; 2.50)		0.81 (−0.38; 2.01)		4.79 (3.51; 6.06)		3.05 (1.81; 4.29)		4.18 (2.22; 6.14)		2.73 (0.64; 4.83)	
Gestational age (weeks)		0.001		0.044		0.136		0.780		0.361		0.575
≥37	Ref		Ref		Ref		Ref		Ref		Ref	
<37	3.18 (1.24; 5.12)		1.98 (0.53; 3.90)		−1.04 (−0.33; 2.41)		−0.20 (−1.62; 1.22)		0.87 (−0.99; 2.73)		0.60 (−1.49; 2.68)	
Type of delivery		<0.001		0.022		<0.001		0.254		0.015		0.269
Vaginal	Ref		Ref		Ref		Ref		Ref		Ref	
Cesarean	−1.79 (−2.68; −0.90)		−1.03 (−1.91; −0.15)		−1.93 (−2.84; −1.02)		−0.51 (−1.38; 0.36)		−1.56 (−2.82; −0.31)		−0.72 (−2.00; 0.56)	
Birth weight (g)		0.064		0.209		0.002		<0.001		0.316		0.038
<2500	1.75 (0.12; 3.39)		1.07 (−0.65; 2.79)		1.75 (0.12; 3.39)		3.83 (2.14; 5.52)		0.85 (−1.58; 3.29)		1.76 (−1.05; 4.57)	
2500–3499	0.39 (−0.48; 1.26)		0.38 (−0.48; 1.23)		0.39 (−0.48; 1.26)		1.52 (0.60; 2.44)		0.79 (−0.64; 2.21)		1.64 (0.24; 3.04)	
≥3500	Ref		Ref		Ref		Ref		Ref		Ref	

^a^Mutually adjusted for all variables in the table, using multiple imputations for handling missing data.

^b^In units milli-g (1000 mg = 1 g = 9.81 m/s^2^).

^c^Cohort mean age (SD) in years: 1982 cohort: 30.2 (0.2); 1993 cohort: 18.4 (0.3); 2004 cohort: 6.7 (0.3).

The overall pattern of associations regarding MVPA is similar to that regarding overall physical activity. Generally, males and those with lower family income and maternal education at birth had higher levels of MVPA. Specifically, the average difference in MVPA between sex was around 22 minutes per day in the 1993 and 2004 cohorts and around 13 minutes in the 1982 cohort. On average, those with the lowest maternal education (i.e., zero schooling) had more MVPA than those with the highest maternal education (i.e., 9+ years schooling) by a margin of 13 minutes per day in the 2004 cohort (not statistically significant), 17 minutes in the 1993 cohort and 9 minutes in the 1982 cohort. The association with income was similar, with the difference between the lowest and highest income categories being 7–10 minutes across cohorts. In the 1993 cohort, compared with participants whose mother was normal weight, those whose mother was underweight had around 6 more minutes of MVPA; those with a highest birth order (i.e., 4+) had nearly 8 more minutes of MVPA than firstborns; compared with participants with a birthweight of ≥3500 g, those with a birthweight of <2500 g had 5 more minutes and those with a birthweight of 2500–3499 g had nearly 2 more minutes of MVPA per day. Cesarean delivery was associated with an average of 5 minutes/day less MVPA in the 1982 cohort (Table 3).

**Table 3:** Unadjusted and adjusted associations^a^ between predictors at birth and total moderate-to-vigorous physical activity^b^ in three birth cohorts in Pelotas, Brazil.

Variables	1982 Cohort (n = 2740)^c^				1993 Cohort (n = 3592)^c^				2004 Cohort (n = 2603)^c^			
	Unadjusted β (95% CI)	p	Adjusted β (95% CI)	p	Unadjusted β (95% CI)	p	Adjusted β (95% CI)	p	Unadjusted β (95% CI)	p	Adjusted β (95% CI)	p
Family income (No of minimum wages per month)		<0.001		0.005		<0.001		<0.001		<0.001		<0.001
≤1	14.98 (8.56; 21.41)		7.39 (0.60; 14.19)		17.93 (11.75; 25.10)		9.73 (3.36; 16.10)		10.56 (3.76; 17.37)		8.57 (1.55; 15.58)	
1.1–3.0	9.76 (3.63; 15.90)		4.41 (−1.93; 19.75)		14.26 (8.62; 19.90)		7.79 (2.05; 13.52)		6.51 (0.07; 12.94)		5.06 (−1.52; 11.63)	
3.1–6.0	6.05 (−0.46; 12.55)		2.65 (−3.70; 9.01)		7.93 (1.99; 13.87)		4.01 (−1.80; 9.83)		2.71 (−4.01; 9.44)		1.58 (−5.07; 8.22)	
6.1–10.0	2.80 (−5.03; 10.64)		0.61 (−6.85; 8.06)		3.20 (−3.95;10.34)		2.03 (−4.70; 8.76)		0.76 (−7.45; 8.97)		0.46 (−7.51; 8.42)	
>10	Ref		Ref		Ref		Ref		Ref		Ref	
Maternal education (year of schooling)		<0.001		0.001		<0.001		<0.001		0.152		0.205
0	13.04 (7.30; 18.78)		9.14 (3.03; 15.24)		23.09 (13.57; 32.60)		16.59 (7.37; 25.81)		14.80 (−0.92; 30.51)		13.19 (−1.95; 28.32)	
1–4	10.09 (6.76; 13.42)		5.66 (1.81; 9.51)		12.73 (8.83; 16.62)		6.62 (2.45; 10.79)		4.24 (0.14; 7.13)		0.80 (−3.66; 5.26)	
5–8	7.11 (4.04; 10.18)		3.60 (0.24; 6.96)		10.08 (6.71; 13.45)		5.66 (2.18; 9.13)		5.27 (2.37; 8.17)		3.10 (0.04; 6.16)	
9+	Ref		Ref		Ref		Ref		Ref		Ref	
Sex		<0.001		<0.001		<0.001		<0.001		<0.001		<0.001
Male	Ref		Ref		Ref		Ref		Ref		Ref	
Female	−13.59 (−15.89; −11.29)		−13.49 (−15.73; −11.26)		−29.07 (−31.67; −26.48)		−22.19 (−31.77; −26.61)		−21.84 (−24.37; −19.31)		−21.86 (−24.42; −19.29)	
Pre-pregnancy body mass index (kg/m^2^)		0.451		0.347		0.007		0.045		0.809		0.909
<18.5	3.60 (−1.37; 8.58)		2.38 (−2.15; 6.91)		9.79 (4.79; 14.79)		5.81 (1.17; 10.44)		−6.03 (−13.80; 1.73)		−3.41 (−10.41; 3.59)	
18.5–24.9	Ref		Ref		Ref		Ref		Ref		Ref	
25–29.9	−1.94 (−5.38; 1.49)		−2.00 (−5.13; 1.14)		−0.31 (3.99; 3.37)		−0.09 (−3.53; 3.35)		−0.74 (−4.47; 2.98)		−0.63 (−4.16; 2.89)	
≥30	3.28 (−2.82; 9.37)		1.80 (−3.82; 7.42)		−2.12 (8.64; 4.40)		−2.91 (−9.01; 3.19)		−2.27 (−7.23; 2.70)		−1.02 (−5.95; 3.91)	
Birth order		0.009		0.246		<0.001		0.001		0.274		0.397
1	Ref		Ref		Ref		Ref		Ref		Ref	
2 or 3	1.60 (−0.97; 4.17)		1.52 (−0.96; 4.00)		1.67 (−1.31; 4.66)		1.21 (−1.60; 4.02)		−0.20 (−3.09; 2.69)		0.40 (−2.45; 3.25)	
≥4	4.73 (1.26; 8.21)		1.67 (−1.85; 5.18)		11.94 (7.73; 16.14)		7.73 (3.64; 11.81)		3.13 (−1.07; 7.32)		2.18 (−2.25; 6.61)	
Gestational age (weeks)		0.135		0.240		0.058		0.661		0.095		0.464
≥37	Ref		Ref		Ref		Ref		Ref		Ref	
<37	4.42 (−1.38; 10.22)		3.38 (−2.27; 9.04)		4.35 (−0.15; 8.85)		1.04 (−3.62; 5.71)		3.37 (−0.59; 7.32)		1.65 (−2.77; 6.08)	
Type of delivery		<0.001		<0.001		0.001		0.369		0.241		0.758
Vaginal	Ref		Ref		Ref		Ref		Ref		Ref	
Cesarean	−6.42 (−9.08; −3.77)		−4.62 (−7.21; −2.02)		−4.87 (−7.85; −1.89)		−1.32 (−4.19; 1.56)		−1.60 (−4.28; 1.07)		−0.43 (−3.14; 12.29)	
Birth weight (g)		0.430		0.634		0.024		<0.001		0.441		0.098
<2500	2.31 (−2.57; 7.20)		1.22 (−3.84; 6.28)		7.52 (2.26; 12.78)		10.55 (4.98; 16.11)		3.11 (−2.10; 8.31)		5.05 (−0.91; 11.00)	
2500–3499	0.44 (−2.16; 3.05)		0.41 (−2.11; 2.92)		0.85 (−2.32; 4.02)		3.18 (0.16; 6.21)		−0.15 (−3.19; 2.88)		1.72 (−1.25; 4.69)	
≥3500	Ref		Ref		Ref		Ref		Ref		Ref	

^a^Mutually adjusted for all variables in the table, using multiple imputations for handling missing data. ^b^In units minutes/day. ^**c**^Cohort mean age (SD) in years: 1982 cohort: 30.2 (0.2); 1993 cohort: 18.4 (0.3); 2004 cohort: 6.7 (0.3).

The pattern of associations with sedentary time as the outcome was largely consistent with findings regarding physical activity outcomes, but in the opposite direction. Being male and lower family income and maternal education at birth were consistently predictive of lower sedentary time in all cohorts (Table 4). Specifically, female participants had an average of 5 more minutes of sedentary time per day than male participants in the 2004 cohort and the difference was larger in the 1993 and 1982 cohorts (21 and 15 minutes, respectively). The difference in sedentary time between the lowest and highest maternal education categories was around 43 minutes in both the 2004 and 1993 cohorts and 32 minutes in the 1982 cohort. The difference in sedentary time between the lowest and highest income categories was around 15 minutes in the 2004 cohort, 26 minutes in the 1993 cohort, and 29 minutes in the 1982 cohort. Mothers’ BMI was positively associated with the child’s sedentary time in the 1982 cohort, where those with an underweight mother had lower levels of sedentary time (around 8 minutes per day) and those with overweight or obese mother had higher levels of sedentary time (more than 9 minutes per day) than those whose mother had a normal weight. A higher birth order was associated with lower sedentary time based on unadjusted analysis in all three cohorts but was only significant in the adjusted model in the 1993 cohort, where participants with the highest birth orders (i.e., 4+) had around 15 minutes less of sedentary time than firstborns. Based on unadjusted analysis, cesarean delivery was significantly associated with higher sedentary time in all three cohorts, but only significant in adjusted analysis of the 2004 cohort, where cesarean delivery was associated with 7 additional minutes/day of sedentary time.

**Table 4:** Unadjusted and adjusted associations^a^ between predictors at birth and total sedentary time^b^ in three birth cohorts in Pelotas, Brazil.

Variables	1982 Cohort (n = 2740)^c^				1993 Cohort (n = 3592)^c^				2004 Cohort (n = 2603)^c^			
	Unadjusted β (95% CI)	p	Adjusted β (95% CI)	p	Unadjusted β (95% CI)	p	Adjusted β (95% CI)	p	Unadjusted β (95% CI)	p	Adjusted β (95% CI)	p
Family income (No of minimum wages per month)		<0.001		0.009		<0.001		<0.001		<0.001		0.005
≤1	−65.09 (−82.86; −47.31)		−28.75 (−48.23; −9.27)		−54.09 (−67.12; −41.05)		− 25.94 (−40.16; −11.72)		−24.49 (−39.06; −9.92)		−14.74 (−30.45; 0.97)	
1.1–3.0	−56.48 (−73.43; −39.52)		−28.47 (−46.63; −10.31)		−47.06 (−58.97; −35.15)		−25.63 (−38.43; −12.83)		−17.39 (−31.17; −3.62)		−9.33 (−24.05; 5.39)	
3.1–6.0	−36.21 (−54.21; −18.22)		−19.69 (−37.92; −1.46)		−23.86 (−36.40; 11.32)		−9.84 (−22.82; 3.13)		−8.27 (−22.67; 6.13)		−4.21 (−19.09; 10.66)	
6.1–10.0	−31.87 (−53.65; −10.09)		−23.44 (−44.86; −2.03)		−8.84 (−23.96; 6.27)		−2.63 (−17.63; 12.37)		2.03 (−15.51; 19.59)		2.85 (−14.98; 20.69)	
>10	Ref		Ref		Ref		Ref		Ref		Ref	
Maternal education (year of schooling)		<0.001		<0.001		<0.001		<0.001		<0.001		0.008
0	−41.87 (−57.74; 26.00)		−32.24 (−49.83; −14.65)		−60.41 (−80.40; −40.43)		−43.12 (−63.75; −22.50)		−52.79 (−86.17; −19.40)		−42.50 (−76.33; −8.67)	
1–4	−47.07 (−56.33; −37.82)		−36.12 (−47.20; −25.03)		−51.82 (−60.00; −43.64)		−36.43 (−45.74; −27.13)		−18.39 (−27.10; −9.67)		−9.53 (−19.53; 0.47)	
5–8	−37.21 (−45.74; −28.68)		−28.72 (−38.41; −19.04)		−34.71 (−41.81; −27.61)		−23.38 (−31.14; −15.62)		−13.40 (−19.56; −7.23)		−7.49 (−14.36; −0.62)	
9+	Ref		Ref		Ref		Ref		Ref		Ref	
Sex		<0.001		<0.001		<0.001		<0.001		0.100	<0.001	0.007
Male	Ref		Ref		Ref		Ref		Ref		Ref	
Female	15.21 (8.71; 21.91)		14.96 (8.52; 21.40)		20.29 (14.42; 26.17)		20.64 (14.89; 26.40)		4.77 (−0.92; 10.46)		5.32 (0.43; 11.07)	
Pre-pregnancy body mass index (kg/m^2^)		0.027		0.012		0.691		0.655		0.476		0.401
<18.5	−12.23 (−26.06; 1.60)		−7.50 (−20.57; 5.57)		−9.06 (−19.79; 1.67)		−2.71 (−13.08; 7.67)		16.92 (−0.12; 33.95)		9.15 (−6.24; 24.55)	
18.5–24.9	Ref		Ref		Ref		Ref		Ref		Ref	
25–29.9	7.97 (−1.53; 17.46)		9.29 (0.10; 18.48)		−0.67 (−8.54; 7.21)		−0.62 (−8.30; 7.06)		3.18 (−5.00; 11.36)		0.38 (−7.48; 8.25)	
≥30	3.91 (−12.88; 20.71)		9.66 (−6.62; 25.94)		−0.06 (−14.11; 13.99)		2.80(−10.88; 16.48)		−1.77 (−12.67; 9.13)		−2.70 (−13.57; 8.18)	
Birth order		0.028		0.256		<0.001		0.001		0.002		0.271
1	Ref		Ref		Ref		Ref		Ref		Ref	
2 or 3	−0.55 (−7.78; 6.69)		−0.72 (−7.87; 6.44)		−10.55 (−16.94; −4.17)		−7.66 (−13.94; −1.38)		−2.77 (−8.93; 3.39)		−0.81 (−7.19; 5.57)	
≥4	−12.94 (−22.72; −3.17)		−6.80 (−16.93; 3.33)		−28.75 (−37.73; −19.78)		−15.05 (−24.17; −5.93)		−15.87 (−24.82; −6.92)		−6.67 (−16.60; 3.26)	
Gestational age (weeks)		0.006		0.070		0.316		0.739		0.612		0.948
≥37	Ref		Ref		Ref		Ref		Ref		Ref	
<37	−22.25 (−38.21; −6.29)		−14.83 (−30.89; 1.22)		−4.90 (−14.48; 4.68)		1.78 (−8.72; 12.28)		−2.19 (−10.65; 6.27)		−0.33 (−10.24; 9.58)	
Type of delivery		<0.001		0.068		<0.001		0.146		<0.001		0.021
Vaginal	Ref		Ref		Ref		Ref		Ref		Ref	
Cesarean	16.61 (9.14; 24.08)		6.95 (−0.52; 14.42)		16.02 (9.66; 22.38)		4.75 (−1.65; 11.16)		10.56 (4.86; 16.27)		7.16 (1.09; 13.22)	
Birth weight (g)		<0.001		0.261		<0.001		0.006		0.065		0.080
<2500	−12.18 (−25.92; 1.57)		−2.61 (−17.16; 11.94)		−16.03 (−27.29; −4.78)		−15.81 (−28.29; −3.34)		−8.18 (−19.28; 2.93)		−8.25 (−21.60; 5.08)	
2500–3499	−8.19 (−15.51; −0.87)		−5.72 (−12.97; 1.53)		−7.94 (−14.72; −1.17)		−7.53 (−14.29; −0.77)		−5.71 (−12.19; 0.77)		−5.93 (−12.58; 0.73)	
≥3500	Ref		Ref		Ref		Ref		Ref		Ref	

^a^Mutually adjusted for all variables in the table, using multiple imputations for handling missing data. ^b^In units minutes/day.

^c^Cohort mean age (SD) in years: 1982 cohort: 30.2 (0.2); 1993 cohort: 18.4 (0.3); 2004 cohort: 6.7 (0.3).

## Discussion

Findings from our study extend the current knowledge on early-life predictors of physical activity and sedentary behaviour using population-based data from a low- and middle- income country setting. Of all the prenatal and birth predictors examined, socioeconomic status at birth had the strongest and most consistent associations with physical activity and sedentary time, where children born into families with lower socioeconomic status (measured by both maternal education and family income) later became more physically active and less sedentary from childhood to adulthood. This finding echoed earlier analyses of the 1993 cohort^14,22^, but contradicted the established evidence from high-income countries where people of higher socioeconomic status are more physically active^7^. A systematic review based on studies primarily from high-income countries found a consistent and positive association of childhood socioeconomic status with leisure-time physical activity in adulthood and an inverse association with transport and occupational physical activity^21^. In high-income countries, where leisure-time physical activity accounts for a large proportion of total physical activity, higher socioeconomic status is consistently associated with higher level of overall physical activity^7^. In low- and middle- income countries, such as Brazil, where the majority of physical activity is not volitional in nature^23^, lower socioeconomic status appears consistently associated with higher levels of total and moderate-to-vigorous physical activity. The consistency of associations in all three cohorts from childhood to adulthood may be explained by limited opportunities for social mobility in Brazil^21^; children born into low socioeconomic status households mostly remain disadvantaged as adults and are therefore more likely to work in labor jobs and travel actively, contributing to higher overall levels of physical activity^22^.

Our study found a substantial sex difference in physical activity, persistent from childhood to adulthood. Boys engage in 20+ min/day more MVPA than girls at age 6 and 18 and men did 13 min/day more MVPA than women at age 30. Females also had higher levels of sedentary time: although the sex difference was small at age 6 (5 min/day), it increased to 20 min/day at age 18 and stayed around 15 min/day at age 30. In most countries around the world, women are found to be consistently less active than men^24^. As there is no obvious established biological explanation for the sex difference in physical activity and sedentary time, social and cultural factors may have constrained girls and women from participating in physical activity^25^ and it is important to target women through culturally acceptable large-scale interventions^26^.

Overall, birth and early life predictors of physical activity and sedentary time differ by cohort/life stage. Maternal BMI, gestational age, type of delivery and birth weight were associated with physical activity and sedentary time in some cohorts. Despite statistical significance, the effect sizes were small; and in almost all cases, the effect sizes were substantially attenuated in the adjusted models, suggesting confounding by other predictors, particularly socioeconomic status. Some evidence suggests a weak association between birth weight and physical activity later in life. A recent meta-analysis of 13 Nordic cohorts found an inverse U-shape association between birth weight and leisure-time physical activity where both very low and very high birth weights were associated with lower leisure-time physical activity^20^. A possible explanation is that intrauterine growth restriction compromises physical capacity and motor performance^27^, as extremely low birth weight has been associated with lower muscle mass and muscle strength^28^, developmental coordination disorder^28^ and poorer musculoskeletal and aerobic fitness^27^. Conversely, high birth weight (>4000 g) has been consistently associated with obesity, which may indirectly contribute to lower levels of physical activity and more sedentary time^29^. In the current study, very few participants had extremely low or high birth weight (Supplementary Table 2), which limits the opportunity to examine these aspects here. Our finding linking low birth weights to more physical activity and less sedentary time is unique, and may be explained by potential mediation by adiposity as those with lower birth weight are less likely to become obese later in life^30^, and therefore may consequentially be more physically active and less sedentary^31,32^.

Our study found a potential association between birth order and physical activity, particularly in the 1993 cohort. The pattern of association is consistent: those of higher birth order were more physically active and less sedentary in adolescence. Having siblings provides opportunities for active interactions. Thus those with the higher birth order may benefit from having older siblings to play with^14^. However, another likely explanation is that birth order is a proxy for parity, which is inversely associated with socioeconomic status. Therefore, the association observed between birth order and activity outcomes may result from residual confounding by socioeconomic status. To date, very few studies have examined the association between birth order and physical activity or sedentary behaviour, and the findings have been mixed^10,14,33^. The observed association between birth order and activity outcomes may involve complex socioeconomic, environmental, and behavioural mechanisms. Future studies could benefit from collecting additional information, such as parity, parenting styles, and the physical activity patterns of parents and siblings.

The study used three birth cohorts including nearly all live births in each study year, with very high cohort retention rates in an low- and middle- income country setting^34^. Physical activity and sedentary time were measured objectively using accelerometers following standardized protocols. However, this study is subject to several limitations. First, although physical activity was measured objectively, some potential issues still apply. Accelerometer measures were collected at one time only, which does not allow for modeling long-term behavioral patterns. Physical activity level was assessed based on at least two days of valid wear time. Although including four or more days of accelerometer data may improve the reliability of the activity estimates^35^, for consistency with our previous paper describing the physical activity levels in the three cohorts^36^, we chose to include all individuals providing at least two days of data. However, it is unlikely this will affect the observed associations as the average wear time was high (i.e. 5.0 days, interquartile range 4.6–5.7 days) and did not differ by sex, socioeconomic and weight status^36^. Furthermore, following the previous publication^36^, we used the same intensity thresholds for all three cohorts. While there is currently no consensus regarding age-specific intensity thresholds from raw wrist-worn accelerometry, the patterns of descriptive physical activity data by age are in agreement with hip-worn accelerometry^37^, hence providing face validity to our observations. While including three cohorts with standardized data collection provides opportunities for comparison across cohorts of different ages; interpreting different patterns of associations is limited by the lack of outcome measures across cohorts at a comparable age, as it is impossible to disentangle age and cohort effects. For example, we could not determine whether the findings regarding birth order were specific to the adolescent age or the 1993 cohort. Furthermore, some predictors were measured by mothers’ self-report, such as pre-pregnancy BMI, which are subject to reporting bias. Finally, while data from Brazil fill a major gap in the literature that is currently mainly based on data from high-income countries, the external validity of the study findings is yet to be determined. Given that the confounding patterns of early life predictors in low- and middle- income countries tend to differ from those observed in high-income countries^34^, findings from the Pelotas cohorts should be compared with other cohorts with caution and a clear understanding of the contextual differences. Birth cohort studies, particularly those from low- and middle- income country, should continue to comprehensively examine biological, behavioural, socioeconomic, and environmental influences on physical activity and sedentary time.

In summary, we found sex and socioeconomic status at birth to be the strongest predictors of physical activity and sedentary time later in life, persisting from childhood to adulthood. Low birth weight and high birth order may be associated with more physical activity and less sedentary time in Brazil. These findings should be interpreted within the context of economic development and social transitions in low- and middle- income countries, such as declines in fertility rates and improvement in neonatal health, which may lead to increasingly inactive lifestyles. Public health actions are urgently needed to prevent further declines in physical activity and increases in sedentary time at the population level.

## Methods

Complete cohort profiles describing baseline characteristics and follow-up data collection have been previously published^14,38,39^. Briefly, participants or their legal guardians voluntarily signed a consent letter before participating in each data collection. Prenatal and birth predictors were measured objectively by trained staff or by mother’s self-report. Written informed consent was obtained from all participants or their parents/legal guardians. All study protocols were approved by the Ethics Committee of the Federal University of Pelotas Medical School (affiliated with the Brazilian Federal Medical Council) and all research procedures were conducted following the approved protocols which are in accordance with relevant guidelines and regulations.

### Measures

Maternal information and child anthropometric measures were collected in a standardized manner at baseline and all subsequent follow-ups to enable cross-cohort comparisons. Birth weight was measured using portable pediatric scales that were calibrated weekly by the research team (CMS and SECA) (precision 100 g) and categorized in three groups as follows: <2500 g, 2500–3499 g, and ≥3500 g^14^. Gestational age was estimated based on the last menstrual period reported by the mothers and <37 weeks was defined as preterm^40^. Family income (categorized as ≤1, 1.1–3.0, 3.1–6.0, 6.1–10.0, >10 times the minimum wage), maternal education (years of schooling; categorized as 0, 1–4, 5–8, and 9+), type of delivery (vaginal vs cesarean) and birth order (1, 2 or 3, and ≥4) were reported by mother at baseline. Finally, pre-pregnancy weight and height were reported by mother to calculate BMI; categorised as underweight (<18.5 kg/m^2^), normal weight (18.5–24.9 kg/m^2^), overweight (25–29.9 kg/m^2^) and obese (≥30 kg/m^2^).

Objectively measured physical activity and sedentary time were collected using GENEActiv (ActivInsights, Kimbolton, UK) accelerometers when participants were 30, 18, and 6 years old for the 1982, 1993, and 2004 birth cohorts, respectively. GENEActiv is a waterproof triaxial monitor, which provides raw data acceleration expressed in milli-*g* units (1000 m*g* = 1 *g* = 9.81 m/s^2^), directly sampled from the MEMS chip. Accelerometers were set up to collect data at 85.7 Hz resolution in the +/−8 *g* range using the GENEActiv software.

**Figure 1:**
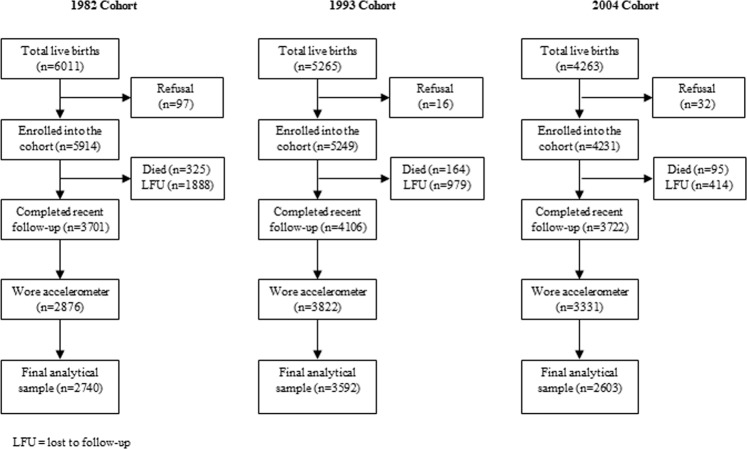
Participants flow.

Participants were invited to wear a wrist-worn accelerometer following a 24 hour-protocol including at least one weekend day. Disabled participants, those who were living outside of Pelotas, those who were unable to wear the accelerometers during work (1982 and 1993 cohorts only), and those who wore a different model of accelerometer that later was terminated unexpectedly (early stage of 2004 cohort only) were excluded from the objective physical activity and sedentary time assessment (Fig. 1). Women who were pregnant were contacted after delivery and invited to wear the accelerometer then. The analytical sample had at least 2 days of valid wear time. The average days of measurement provided by participants was 5.0 (SD = 1.0) and there was no difference in wear time by sex, socioeconomic or weight status^36^. Further details and the complete research protocol are available elsewhere^41^.

The R-package GGIR (http://cran.r-project.org) was used to analyze binary files from the GENEActiv accelerometers. A detailed signal-processing scheme and data reduction process have been described elsewhere^42^. In brief, three summary variables were created: overall physical activity (milli-g), time spent in (min/day) MVPA, and sedentary time (min/day). Overall physical activity is an estimate of the total volume of movement expressed by the average vector magnitude of wrist acceleration per day. MVPA was estimated as the time spent in 10-min bouts in which at least 80% of each bout was spent with accelerations equal to or higher than 100 m*g*^36^. The assessment of sedentary time was restricted to an expected awake period arbitrarily defined between 7 am and 11 pm. Sedentary time was defined as non-bouted intensities lower than 50 m*g*^43^.

### Data analysis

Analyses were performed using STATA software 14.2 (College Station, TX). Descriptive statistics were presented for each cohort and further compared between the baseline sample at recruitment and the analytical sample. For each outcome, we fitted general linear regression models to examine the unadjusted and adjusted associations (accounting for all other predictors in the model) between prenatal/birth predictors and a physical activity/sedentary time outcome. Results are presented as regression coefficients (β) and 95% CIs representing the average difference in units of each outcome by levels of exposure. Missing data (ranging from 0.01% to 14.4%) on independent variables were handled using multiple imputation with all independent variables and the outcome variable included in each imputation model and 100 imputations (m = 100)^44^.

### Ethical approval

Federal University of Pelotas Medical School ethics committee, affiliated with the Brazilian Federal Medical Council.

## Supplementary information

Supplementary files.
