# Supplementary material for: Prenatal and birth predictors of objectively measured physical activity and sedentary time in three population-based birth cohorts in Brazil
Source: Sci Rep. 2020 Jan 21;10:786. doi: 10.1038/s41598-019-57070-x (PMC6972919; doi:10.1038/s41598-019-57070-x)
Supplement: Supplementary file 1 — Supplementary files. [file 41598_2019_57070_MOESM1_ESM.pdf]

Prenatal and birth predictors of objectively measured physical activity and sedentary time in three population-based birth cohorts in Brazil

Ding Ding*, PhD;[1,2] Gregore I. Mielke, PhD;[3,4] Inacio de Silva, PhD;[4] Fernando C. Wehrmeister, PhD;[4,5] Bernardo L. Horta, PhD;[4,5] Soren Brage, PhD;[6] Pedro C. Hallal, PhD;[4] Ulf Ekelund, PhD[7]

----Supplementary files---

Supplementary Table 1. Comparison of characteristics between the original and analytical samples (1982, 1993, 2004 birth cohorts, Pelotas, Brazil)

Characteristics	1982		1993		2004	
	Original (n=5914)* n (%)	Analytical (n=2740)* n (%)	Original (n=5249)* n (%)	Analytical (n=3592)* n (%)	Original (n=4231)* n (%)	Analytical (n=2603)* n (%)
Sex						
Male	3037 (51.4)	1329 (48.5)	2606 (49.7)	1764 (49.1)	2196 (51.9)	1341 (51.5)
Female	2876 (48.6)	1411 (51.5)	2632 (50.3)	1828 (50.9)	2035 (48.1)	1262 (48.5)
Birth weight (g)						
< 2500	534 (9.0)	196 (7.2)	510 (9.8)	331 (9.2)	424 (10.0)	224 (8.6)
2500-3499	3613 (61.2)	1711 (62.4)	3361 (64.2)	2285 (63.7)	2694 (63.7)	1663 (63.9)
≥ 3500	1762 (29.8)	832 (30.4)	1361 (26.0)	972 (27.1)	1110 (26.3)	716 (27.5)
Gestational age (weeks)						
< 37	294 (6.3)	120 (5.5)	611 (11.8)	381 (10.7)	613 (14.5)	338 (13.0)
≥ 37	4380 (93.7)	2075 (94.5)	4582 (88.2)	3171 (89.3)	3604 (85.5)	2263 (87.0)
Family income (No of minimum wages per month)						
≤ 1	1288 (21.9)	588 (21.6)	967 (18.4)	636 (17.1)	884 (20.9)	524 (20.1)
1.1-3.0	2789 (47.4)	1383 (50.7)	2260 (43.1)	1557 (43.4)	1939 (46.0)	1204 (46.3)
3.1-6.0	1091 (18.5)	505 (18.5)	1204 (22.9)	866 (24.1)	945 (22.4)	599 (23.0)
6.1-10.0	382 (6.5)	141 (5.2)	433 (8.3)	286 (8.0)	243 (5.8)	154 (5.9)
> 10	335 (5.7)	109 (4.0)	385 (7.3)	247 (6.9)	207 (4.9)	121 (4.7)
Maternal education (years of schooling)						
0	327 (5.5)	140 (5.1)	134 (2.6)	85 (2.4)	43 (1.0)	19 (0.7)
1-4	1633 (27.7)	791 (28.9)	1338 (25.5)	879 (24.5)	612 (14.6)	365 (14.2)
5-8	2454 (41.5)	1215 (44.4)	2424 (46.2)	1716 (47.8)	1731 (41.3)	1092 (42.4)
9 +	1493 (25.3)	590 (21.6)	1350 (25.7)	909 (25.3)	1802 (43.0)	1100 (42.7)
Pre-pregnancy body mass index (kg/m^2)						
< 18.5	388 (7.8)	176 (7.5)	451 (8.9)	309 (8.8)	128 (4.3)	78 (4.3)
18.5-24.9	3492 (70.2)	1625 (69.5)	3507 (68.8)	2373 (67.8)	1766 (59.6)	1071 (59.2)
25-29.9	881 (17.7)	421 (18.0)	894 (17.5)	645 (18.4)	738 (24.9)	446 (24.7)
≥ 30	215 (4.3)	116 (5.0)	245 (4.8)	172 (4.9)	333 (11.2)	213 (11.8)
Birth order						
1	2322 (39.3)	1080 (39.4)	2102 (40.1)	1426 (39.7)	1834 (44.2)	1159 (45.3)
2 or 3	2626 (44.4)	1218 (44.5)	2366 (45.1)	1635 (45.6)	1738 (41.9)	1060 (41.4)
≥ 4	964 (16.3)	441 (16.1)	776 (14.8)	528 (14.7)	574 (13.8)	339 (13.3)

*Numbers vary owing to missing values

Supplementary Table 2. Distribution of birth weight in all three cohorts in Pelotas, Brazil

Birth weight (g)	1982 cohort	1993 cohort	2004 cohort
	n (%)	n (%)	n (%)
<750	10 (0.2)	5 (0.1)	14 (0.3)
751-1250	28 (0.5)	23 (0.4)	30 (0.7)
1251-1750	76 (1.3)	50 (1.0)	43 (1.0)
1751-2250	197 (3.3)	192 (3.7)	153 (3.6)
2251-2750	783 (13.3)	775 (14.8)	589 (13.9)
2751-3250	2090 (35.4)	1900 (36.4)	1538 (36.4)
3251-3750	1953 (33.1)	1673 (32.0)	1376 (32.5)
3751-4250	654 (11.1)	527 (10.1)	430 (10.2)
4251-4750	100 (1.7)	70 (1.3)	49 (1.2)
4751-5250	16 (0.3)	9 (0.2)	4 (0.09)
5251-5750	1 (0.02)	2 (0.04)	1 (0.02)
>5750	1 (0.02)	0 (0)	1 (0.02)

Supplementary information- STROBE Statement—Checklist of items that should be included in reports of ***cohort studies***

	Item No	Recommendation
**Title and abstract**	1	(*a*) Indicate the study's design with a commonly used term in the title or the abstract – *In both the title and the abstract we explicitly mentioned it is a prospective birth cohort study.*
		(*b*) Provide in the abstract an informative and balanced summary of what was done and what was found – *Clearly stated in abstract.*
**Introduction**		
Background/rationale	2	Explain the scientific background and rationale for the investigation being reported – -- *Clearly stated in the Introduction*
Objectives	3	State specific objectives, including any prespecified hypotheses – *Objective stated at the end of the Introduction*
**Methods**		
Study design	4	Present key elements of study design early in the paper — METHODS -- *Stated at the start of Methods*
Setting	5	Describe the setting, locations, and relevant dates, including periods of recruitment, exposure, follow-up, and data collection – *Stated in Methods*
Participants	6	(*a*) Give the eligibility criteria, and the sources and methods of selection of participants. Describe methods of follow-up – *eligibility criteria, sources and methods of selection were stated in Sampling and Procedures; Methods of follow-up stated in Measures*
		(*b*) For matched studies, give matching criteria and number of exposed and unexposed – *NA*
Variables	7	Clearly define all outcomes, exposures, predictors, potential confounders, and effect modifiers – *Clearly defined in Methods and Table 1* Give diagnostic criteria, if applicable – *NA*
Data sources/ measurement	8*	For each variable of interest, give sources of data and details of methods of assessment (measurement) – *References for measures provided whenever possible* Describe comparability of assessment methods if there is more than one group – *NA*
Bias	9	Describe any efforts to address potential sources of bias – *Participants with and without accelerometer data were compared (Appendix Table 1) sensitivity analysis conducted using different missing data handling*
Study size	10	Explain how the study size was arrived at – *Stated in Results and presented in Figure 1*
Quantitative variables	11	Explain how quantitative variables were handled in the analyses. If applicable, describe which groupings were chosen and why – *Stated in Methods (Measures and Statistical Analysis)*
Statistical methods	12	(*a*) Describe all statistical methods, including those used to control for confounding – *Stated in Methods (Statistical Analysis)*
		(*b*) Describe any methods used to examine subgroups and interactions – *Stated in Methods (Statistical Analysis)*
		(*c*) Explain how missing data were addressed – *Discussed in Methods (Measures and Statistical Analysis)*
		(*d*) If applicable, explain how loss to follow-up was addressed – *NA*

		(*e*) Describe any sensitivity analyses – *Sensitivity analysis conducted as stated in Statistical Analysis and Results*
**Results**		
Participants	13*	(a) Report numbers of individuals at each stage of study—eg numbers potentially eligible, examined for eligibility , confirmed eligible , included in the study, completing follow-up , and analysed – *Presented in Figure 1*
		(b) Give reasons for non-participation at each stage –*Presented in Figure 1*
		(c) Consider use of a flow diagram – *Figure 1*
Descriptive data	14*	(a) Give characteristics of study participants (eg demographic, clinical, social) and information on exposures and potential confounders – *Stated in Results (Descriptive Statistics) and Table 1*
		(b) Indicate number of participants with missing data for each variable of interest – *Indicated in Appendix Table 2*
		(c) Summarise follow-up time (eg, average and total amount) – *Indicated in Results (Descriptive Statistics)*
Outcome data	15*	Report numbers of outcome events or summary measures over time – *Summary of physical activity measures are indicated in Results and Table 1*
Main results	16	(*a*) Give unadjusted estimates and, if applicable, confounder-adjusted estimates and their precision (eg, 95% confidence interval). Make clear which confounders were adjusted for and why they were included – *Indicated in Results , Table 2-4*
		(*b*) Report category boundaries when continuous variables were categorized – *Indicated in all tables*
		(*c*) If relevant, consider translating estimates of relative risk into absolute risk for a meaningful time period – *We reported beta coefficients which represent the difference in outcome values between categories of exposure.*
Other analyses	17	Report other analyses done—eg analyses of subgroups and interactions, and sensitivity analyses – *Included in appendix analysis. Test of interaction conducted and reported*
**Discussion**		
Key results	18	Summarise key results with reference to study objectives – *Addressed*
Limitations	19	Discuss limitations of the study, taking into account sources of potential bias or imprecision. Discuss both direction and magnitude of any potential bias – *Extensively described in Discussion (Strengths and Limitations)*
Interpretation	20	Give a cautious overall interpretation of results considering objectives, limitations, multiplicity of analyses, results from similar studies, and other relevant evidence – *Cautious interpretation provided in Discussion*
Generalisability	21	Discuss the generalisability (external validity) of the study results – *Addressed in Discussion*
**Other information**		
Funding	22	Give the source of funding and the role of the funders for the present study and, if applicable, for the original study on which the present article is based – *Funding and the role of the funder presented.*

*Give information separately for exposed and unexposed groups.

Note: An Explanation and Elaboration article discusses each checklist item and gives methodological background and published examples of transparent reporting. The STROBE checklist is best used in conjunction with this article (freely available on the Web sites of PLoS Medicine at http://www.plosmedicine.org/, Annals of Internal Medicine at

http://www.annals.org/, and Epidemiology at http://www.epidem.com/). Information on the STROBE Initiative is available at http://www.strobe-statement.org.
